# Pediatric Flexor Pollicis Longus Injury: Practical Modifications of Assessment and Repair

**Published:** 2016-07-06

**Authors:** Mark Gorman, Julia Ruston, John Dickson

**Affiliations:** ^a^Division of Plastic Surgery, Queen Victoria Hospital, East Grinstead, West Sussex, United Kingdom; ^b^Division of Plastic Surgery, Broomfield Hospital, Chelmsford, Essex, United Kingdom; ^c^Division of Plastic Surgery, Royal Free Hospital, London, United Kingdom

**Keywords:** flexor pollicis longus, pediatric, assessment, Kapandji scale

Dear Sir,

Pediatric flexor pollicis longus (FPL) injury is an uncommon entity with scarce literature pertinent to the results of repair. Although much has been inferred from the study of long flexor tendons of the other 4 digits, the FPL's unique anatomy has been cited as justification for its independent study. The largest case series of 16 patients has reported outcome analysis using a modification of the otherwise complex Buck-Gramcko and Tubiana assessment scales.^1^^-^3 Such outcome assessment employing multiple joint angle measurement may not be practical in young children and place little emphasis on functional assessment. The Kapandji scale of thumb opposition assesses functional mobility of the thumb, but it can be difficult to employ in noncompliant pediatric patients.^4^

In a quest to propose a more practical pediatric assessment tool for FPL repair outcomes, we conducted a retrospective review of our practice. Seven children ranging in age from 9 months (Fig 1) to 14 years underwent primary repair of complete FPL lacerations (1 zone 1, 5 zone 2, and 1 zone 3) between 2000 and 2010 at our unit. A standard management protocol was observed in this cohort with the primary repair within 48 hours of injury, using a modified 4-strand Kessler core and Silfverskiöld epitendinous suture technique. Postoperative rehabilitation included 4 weeks of boxing glove/splint immobilization for those younger than 8 years and early active mobilization for older children. Patients received an average of 6 to 8 weeks of hand therapy and were followed up for a minimum of 3 months thereafter (mean = 9 months).

Review of the hand therapy assessment charts showed challenges faced by both clinicians and hand therapists with documenting the complex joint measurements required by the existent assessment scales. We thus collaborated with specialist hand therapists to develop a functional assessment scale for more reliable and practical documentation of outcome measures in the pediatric population.

The 10-point Kapandji scale of thumb opposition was simplified to include 3 of its reference points (Fig 1) and then combined with simple functional assessments such as holding a beaker (assessing extension deficit) or picking up a flat coin from a surface (assessing compound flexion)—summarized in Figure 2. Functional mobility of the thumb is a product of compound movements and may be achieved despite individual joints having relatively little movement, leading previous criteria to wrongly determine a poor outcome. Similarly, individuals may achieve good or excellent Kapandji scores despite poor mobility at joints (due to thumb “creep” across the palm) and thus outcomes were validated only in conjunction with functional assessment. The proposed modified Kapandji scale was based on a 4-point scale to be directly comparable with that of Buck-Gramcko, such that a Kapandji score of 9 was rated as excellent, 7 as good, 3 as fair, and any significant functional or extension deficit was automatically graded as poor.

The hand therapy chart notes at 3 months after surgical repair were extrapolated into the assessment proposed by our scale. We did not observe any postoperative tendon ruptures in our series. Four repair outcomes were graded as excellent, 2 as good, and 1 as fair in a year-old boy who required tenolysis following suspected rupture. This was consistent with the functional outcome seen on long-term follow-up of the patients, demonstrating our modified scale to be both practical and reliable in our small cohort of patients. Our preferred outcome assessment enables hand therapists and doctors evaluating repairs in clinic to use the hand as its own frame of reference. The use of stickers on Kapandji reference points and everyday props in functional assessment make this an effective assessment tool that is both child- and user-friendly.

## Figures and Tables

**Figure 1:**
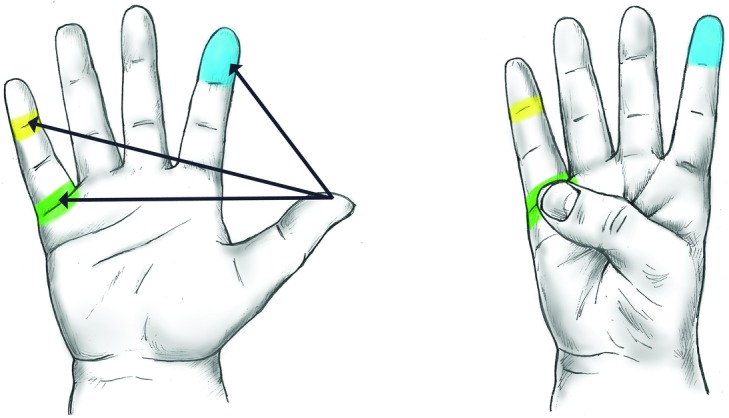
Pictorial illustration of the proposed modified Kapandji scale. The 3 key reference point of thumb.

**Figure 2:**
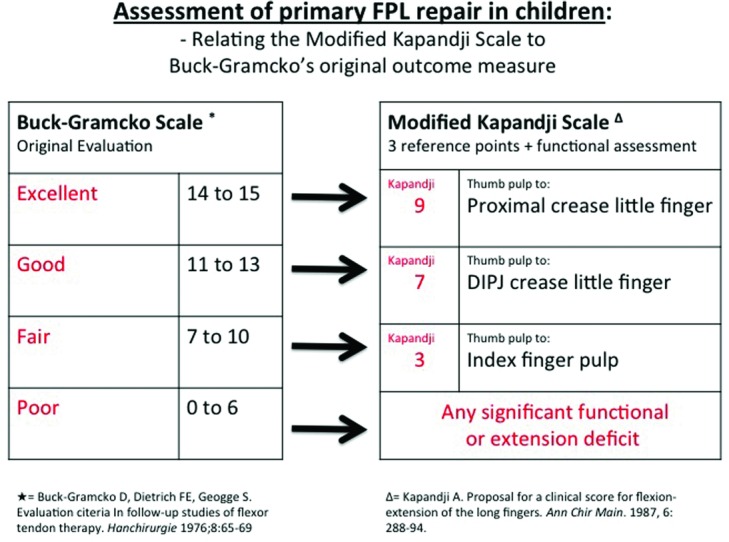
The practical modification of the Buck-Gramcko and Kapandji scales.
